# Close linkage between blood total ketone body levels and B-type natriuretic peptide levels in patients with cardiovascular disorders

**DOI:** 10.1038/s41598-021-86126-0

**Published:** 2021-03-22

**Authors:** Yusuke Kashiwagi, Tomohisa Nagoshi, Yasunori Inoue, Yoshiro Tanaka, Hirotake Takahashi, Yuhei Oi, Haruka Kimura, Kousuke Minai, Michihiro Yoshimura

**Affiliations:** Division of Cardiology, Department of Internal Medicine, The Jikei University School of Medicine, 3-25-8, Nishi-shimbashi, Minato-ku, Tokyo, 105-8461 Japan

**Keywords:** Cardiovascular diseases, Heart failure

## Abstract

In patients with cardiovascular disorders, blood total ketone body (TKB) levels increase with worsening heart failure and are consumed as an alternative fuel to fatty acid and glucose. We investigated factors contributing to the increase in the blood TKB levels in patients with cardiovascular disorders. The study population consisted of 1030 consecutive patients who underwent cardiac catheterization. Covariance structure analyses were performed to clarify the direct contribution of hemodynamic parameters, including the left ventricular end-diastolic pressure (LVEDP), left ventricular end-systolic volume index (LVESVI), left ventricular end-diastolic volume index (LVEDVI), and B-type natriuretic peptide (BNP) levels, to TKB by excluding other confounding factors. These analyses showed that the TKB levels were significantly associated with the BNP level (*P* = 0.003) but not the LVEDP, LVESVI, or LVEDVI levels. This was clearly demonstrated on a two-dimensional contour line by Bayesian structure equation modeling. The TKB level was positively correlated with the BNP level, but not LVEDP, LVESVI or LVEDVI. These findings suggested that elevated blood TKB levels were more strongly stimulated by the increase in BNP than by hemodynamic deterioration. BNP might induce the elevation of TKB levels for use as an important alternative fuel in the failing heart.

## Introduction

Derangement in myocardial fuel metabolism contributes to the development of heart disease. In the normal heart, cardiac energy metabolism mainly depends on fatty acids and glucose^1,2^. Although ketone bodies are a minor energy substrate in the normal heart, the elevation of blood ketone body levels provides an important alternative energy substrate in the failing heart^3–5^. The utilization of ketone bodies in extrahepatic tissues such as the heart was determined by circulating ketone body levels^6–8^. In order to clarify the factors that increase the utilization of ketone bodies in the heart, it is necessary to show the factors that elevate blood ketone body levels. However, it was reported that blood ketone body levels in patients with heart failure were elevated in proportion to the severity of their symptoms and cardiac dysfunction as well as the degree of activation of stress hormones^9^, and the detailed mechanism is not fully understood.

Although there are various indicators of heart failure, the left ventricular end-diastolic pressure (LVEDP), left ventricular end-systolic volume index (LVESVI), left ventricular end-diastolic volume index (LVEDVI), and the plasma level of B-type natriuretic peptide (BNP) are the main indicators of the hemodynamics and the severity of heart failure^10–12^. In particular, the BNP level is considered a sensitive and reliable biomarker of the degree of heart failure in clinical practice^13^. Natriuretic peptides (NPs) are produced in the heart and classically affect the renal and cardiovascular systems. The major physiological effects of NPs are natriuresis, vasodilation, and inhibition of the renin–angiotensin–aldosterone (RAA) and sympathetic nervous systems^14,15^. However, it is currently believed that NPs have a variety of other effects. Recently, many researchers have focused on the relationship between NPs and energy metabolism^16–18^, and we reported several investigations about this relationship^19–23^. However, in patients with cardiovascular disorders, the direct relationship between NPs and ketone bodies has not been fully elucidated.

In patients with heart failure, due to various confounding factors, such as comorbid disease (e.g. diabetes) and oral medicine use^24^, it is difficult to clarify the precise factors that affect the blood total ketone body (TKB) level. In the present study, we performed a covariance structure analysis to clarify the factors that contribute to the increase in blood ketone body levels in patients with cardiovascular disorders.

## Results

### Patient characteristics

Table 1 shows the clinical characteristics of the patients in this study. The median BNP level was 47.8 pg/mL (interquartile range [IQR] 19.6–131 pg/mL), the median LVEDP was 11.0 mmHg (IQR 8.0 -14.0 mmHg), the median LVESVI was 23.6 mL/m^2^ (IQR 17.7–33.5 mL/m^2^), the median LVEDVI was 61.5 mL/m^2^ (IQR 50.5–74.5 mL/m^2^), and the median TKB level was 194 μmol/L (IQR 89.0–404 μmol/L).

**Table 1:** The clinical characteristics of the patients (n = 1030).

Male (%)	81.4
Age (years)	69.0 (60.0, 76.0)
BMI (kg/m^2^)	24.2 ± 3.9
Mean blood pressure (mmHg)	94.4 ± 15.9
Heart rate (beats per minutes)	69.0 (62.0, 80.0)
Hb (g/dL)	13.5 ± 1.94
eGFR (mL/min/1.73 m^2^)	67.8 (55.4, 80.2)
Total Bilirubin (mg/dL)	0.8 (0.6, 1.0)
Uric Acid (mg/dL)	6.0 ± 1.5
CRP (mg/dL)	0.08 (0.04, 0.24)
HDL (mg/dL)	49.0 (40.3, 60.0)
LDL (mg/dL)	97.3 ± 28.5
Triglyceride (mg/dL)	102 (75, 137)
HbA1c (%)	6.0 (5.6, 6.6)
Fasting blood sugar (mg/dL)	103 (94, 123)
BNP (pg/mL)	47.8 (19.6, 131)
LVEDP (mmHg)	11.0 (8.0, 14.0)
LVESVI (mL/m^2^)	23.6 (17.7, 33.5)
LVEDVI (mL/m^2^)	61.5 (50.5, 74.5)
Total ketone body (μmol/L)	194 (89.0, 404)
**Underlying main cardiovascular disease (%)**	
Ischemic heart disease	74
Valvular disease	9.8
Cardiomyopathy	7.9
Arrhythmia	2.1
Macrovascular disease	0.5
Congenital heart disease	0.3
Constrictive pericarditis	0.3
Other diseases	5.1

*BMI* body mass index, *Hb* hemoglobin, *eGFR* estimated glomerular filtration rate, *CRP* C-reactive protein, *HDL* high-density lipoprotein cholesterol, *LDL* low-density lipoprotein cholesterol, *BNP* B-type natriuretic peptide, *LVEDP* left ventricular end-diastolic pressure, *LVESVI* left ventricular end-systolic volume index, *LVEDVI* left ventricular end-diastolic volume index.

### The correlation of blood TKB levels with various clinical factors

Table 2 shows the Spearman rank correlation coefficients between the TKB level and various clinical factors. The TKB level was significantly and positively correlated with the age, total bilirubin (TB), fasting blood sugar (FBS), hemoglobin A1c (HbA1c), high-density lipoprotein cholesterol (HDL), and BNP and negatively correlated with the sex, body mass index (BMI), mean blood pressure (BP), hemoglobin (Hb), estimated glomerular filtration rate (eGFR), and triglyceride (TG) level.

**Table 2:** Spearman’s rank correlation coefficients between the total ketone body level and various clinical factors (n = 1030).

	R	*P*
Male	− 0.064	0.040
Age	0.170	< 0.001
BMI	− 0.129	< 0.001
Mean blood pressure	− 0.073	0.023
Heart rate	0.088	0.082
Hb	− 0.116	< 0.001
eGFR	− 0.089	0.004
Total Bilirubin	0.097	0.010
Uric acid	− 0.025	0.426
CRP	0.059	0.059
Fasting blood sugar	0.067	0.032
HbA1c	0.091	0.004
HDL	0.095	0.002
LDL	0.057	0.067
Triglyceride	− 0.314	< 0.001
BNP	0.209	< 0.001
LVEDP	0.019	0.583
LVESVI (mL/m^2^)	0.044	0.233
LVEDVI (mL/m^2^)	0.057	0.120

*BMI* body mass index, *Hb* hemoglobin, *eGFR* estimated glomerular filtration rate, *CRP* C-reactive protein, *HDL* high-density lipoprotein cholesterol, *LDL* low-density lipoprotein cholesterol, *BNP* B-type natriuretic peptide, *LVEDP* left ventricular end-diastolic pressure, *LVESVI* left ventricular end-systolic volume index, *LVEDVI* left ventricular end-diastolic volume index.

### The concept and results of the proposed path model (A)

**Figure 1:**
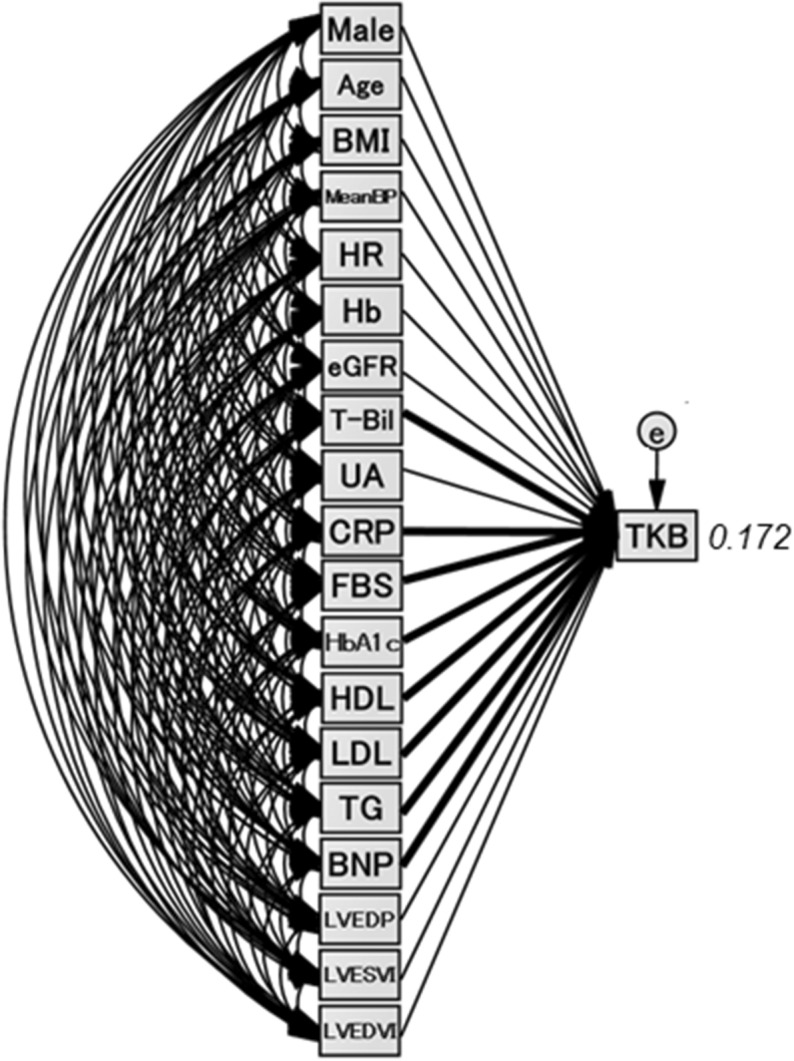
Path model (A). The path model theoretically proposed to clarify the contribution of each of the factors which are show in Table 2 to TKB. Each path has a coefficient representing the standardized coefficient of a regressing independent variable on a dependent variable of the relevant path. These variables represent the standardized regression coefficients (direct effect) (shown in Table 3) and squared multiple correlations (in narrow italics). *BMI* body mass index, *Mean BP* mean blood pressure, *HR* heart rate, *Hb* hemoglobin, *eGFR* estimated glomerular filtration rate, *T-Bil* total-bilirubin, *UA* uric acid, *CRP* C-reactive protein, *FBS* fasting blood sugar, *HDL* high-density lipoprotein cholesterol, *LDL* low-density lipoprotein cholesterol, *TG* triglyceride, *BNP* B-type natriuretic peptide, *LVEDP* left ventricular end-diastolic pressure, *LVESVI* left ventricular end-systolic volume index, *LVEDVI* left ventricular end-diastolic volume index.

The theoretical path model (A) is shown in Fig. 1. Paths were drawn from independent to dependent variables, with directional arrows for each regression model. The association between two factors was linked by two-way arrows. This approach was employed to investigate the factors that exerted causative effects on the blood TKB level. The path model (A) was proposed by positioning the blood TKB level in parallel with the other factors, which are shown in Table 2. The precise results of the path model (A) are shown in Table 3. The path model (A) revealed that the TB, CRP, FBS, HbA1c, HDL, low-density lipoprotein cholesterol (LDL), TG, and BNP values were significantly associated with the blood TKB level, while the LVEDP, LVESVI, and LVEDVI were not.

**Table 3:** The results of the path model (A).

	Estimate	Standard error	Test statistic	*P* value	Standardized regression coefficient
					Direct effect
**TKB (R**^**2**^** = 0.172)**					
Male	0.919	26.238	0.035	0.972	0.001
Age	− 0.045	0.982	− 0.045	0.964	− 0.002
BMI	− 3.596	2.906	− 1.237	0.216	− 0.044
Mean blood pressure	− 0.925	0.653	− 1.416	0.157	− 0.047
Heart rate	0.204	1.140	0.179	0.858	0.009
Hb	− 11.692	6.541	− 1.788	0.074	− 0.072
eGFR	0.652	0.559	1.167	0.243	0.041
Total Bilirubin	63.300	31.417	2.015	0.044	0.076
Uric acid	11.353	6.588	1.723	0.085	0.056
CRP	55.949	8.951	6.250	< 0.001	0.192
Fasting blood sugar	− 0.962	0.405	− 2.374	0.018	− 0.091
HbA1c	50.344	13.868	3.630	< 0.001	0.141
HDL	1.816	0.710	2.558	0.011	0.086
LDL	1.053	0.339	3.111	0.002	0.096
Triglyceride	− 0.930	0.173	− 5.364	< 0.001	− 0.175
BNP	0.202	0.060	3.350	< 0.001	0.172
LVEDP	1.487	2.199	0.676	0.499	0.026
LVESVI	− 0.216	1.422	− 0.152	0.879	− 0.018
LVEDVI	− 0.115	1.160	− 0.099	0.921	− 0.011

*R*^*2*^ squared multiple correlation, *TKB* total ketone body, *BMI* body mass index, *Hb* hemoglobin, *eGFR* estimated glomerular filtration rate, *CRP* C-reactive protein, *HDL* high-density lipoprotein cholesterol, *LDL* low-density lipoprotein cholesterol, *BNP* B-type natriuretic peptide, *LVEDP* left ventricular end-diastolic pressure, *LVESVI* left ventricular end-systolic volume index, *LVEDVI* left ventricular end-diastolic volume index.

### The concept and results of the proposed path model (B)

**Figure 2:**
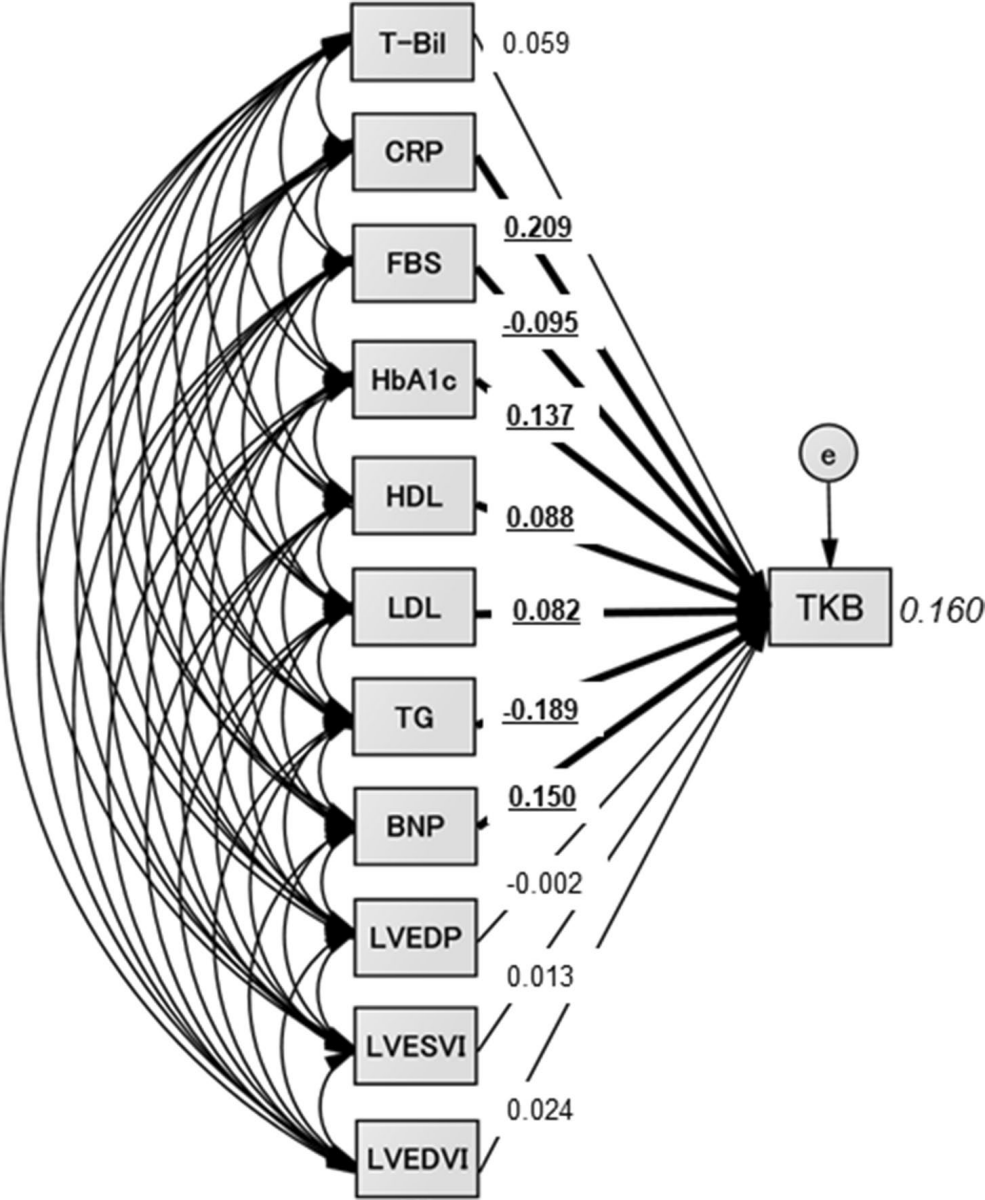
Path model (B). The path model theoretically proposed to clarify the contribution of each factor (T-Bil, CRP, FBS, HbA1c, HDL, LDL, TG, BNP, LVEDP, LVESVI, and LVEDVI) to TKB. Each path has a coefficient representing the standardized coefficient of a regressing independent variable on a dependent variable of the relevant path. These variables represent the standardized regression coefficient (direct effect) (underlined portions indicate remarkable values) and squared multiple correlation (in narrow italics). *T-Bil* total-bilirubin, *CRP* C-reactive protein, *FBS* fasting blood sugar, *HDL* high-density lipoprotein cholesterol, *LDL* low-density lipoprotein cholesterol, *TG* triglyceride, *BNP* B-type natriuretic peptide, *LVEDP* left ventricular end-diastolic pressure, *LVESVI* left ventricular end-systolic volume index, *LVEDVI* left ventricular end-diastolic volume index.

The next theoretical path model (B) is shown in Fig. 2. The path model (B) was proposed by positioning the blood TKB level in parallel with the factors that have been identified as statistically significant according to the path model (A) and the hemodynamic indices (LVEDP, LVESVI, and LVEDVI). The precise results of path model (B) are shown in Table 4. Path model (B) revealed that the CRP, FBS, HbA1c, HDL, LDL, TG, and BNP values were significantly associated with the blood TKB level, while the LVEDP, LVESVI, and LVEDVI were not.

**Table 4:** The results of path model (B).

	Estimate	Standard error	Test statistic	*P* value
**TKB (R**^**2**^** = 0.160)**				
Total Bilirubin	49.408	29.237	1.690	0.091
CRP	60.731	8.735	6.952	< 0.001
Fasting blood sugar	− 1.004	0.400	− 2.513	0.012
HbA1c	48.837	13.819	3.534	< 0.001
HDL	1.845	0.664	2.777	0.005
LDL	0.908	0.331	2.744	0.006
Triglyceride	− 1.005	0.167	− 6.023	< 0.001
BNP	0.176	0.059	2.976	0.003
LVEDP	− 0.125	2.042	− 0.061	0.951
LVESVI	0.153	1.317	0.116	0.908
LVEDVI	0.253	1.077	0.235	0.815

*R*^*2*^ squared multiple correlation, *TKB* total ketone body, *CRP* C-reactive protein, *HDL* high-density lipoprotein cholesterol, *LDL* low-density lipoprotein cholesterol, *BNP* B-type natriuretic peptide, *LVEDP* left ventricular end-diastolic pressure, *LVESVI* left ventricular end-systolic volume index, *LVEDVI* left ventricular end-diastolic volume index.

### The results of Bayesian structural equation modeling

**Figure 3:**
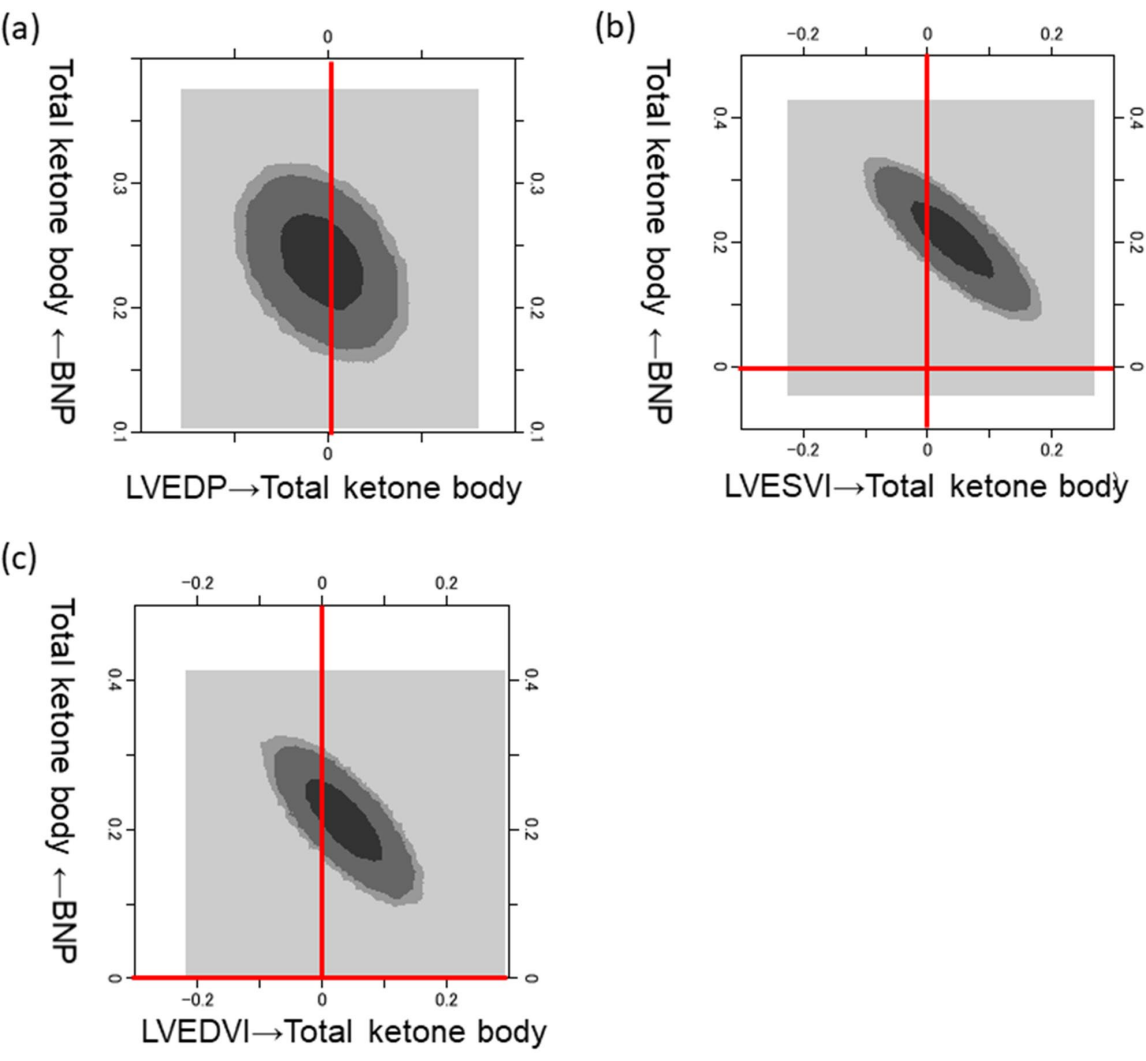
Bayesian structure equation modeling. Frequency polygons were described by the marginal posterior distributions of the estimates. The two-dimensional plot of the bivariate posterior density shows the relationship between the bivariate marginal posterior plots. From light to dark, the 3 shades of gray represent 50%, 90%, and 95% reliable regions, respectively. *BNP* B-type natriuretic peptide, *LVEDP* left ventricular end-diastolic pressure, *LVESVI* left ventricular end-systolic volume index, *LVEDVI* left ventricular end-diastolic volume index.

A two-dimensional (2-D) contour image created by Bayesian structural equation modeling showed that the blood TKB level was positively correlated with the BNP level, while the LVEDP, LVESVI, and LVEDVI were not (Fig. 3).

### The relationship between TKB levels and various clinical factors in the two groups (stratifying patients according to BNP levels)

Next, the patients were divided into 2 groups according to whether the BNP level was normal (≤ 18.4 pg/mL) (n = 244) or high (> 18.4 pg/mL) (n = 786), and the same analyses as shown in path model (A), (B), and the Bayesian structural equation modeling (Fig. 3) were performed in each group. Not only in the high BNP level group but also in the normal BNP level group, similar to the results of previous analyses, the blood TKB level was positively correlated with the BNP level, while the LVEDP, LVESVI, and LVEDVI were not (Supplementary Figure S1-6, Supplementary Table S1-4).

## Discussion

A previous report demonstrated that blood TKB levels increase in proportion to the severity of heart failure, as indicated by the pulmonary artery wedge pressure (PAWP) and left ventricular ejection fraction (LVEF)^9^. The present study showed that blood TKB levels were positively associated with the BNP levels, rather than the hemodynamic indices, such as LVEDP, LVESVI and LVEDVI.

The blood TKB levels were positively correlated with the BNP levels rather than the LVEDP, LVESVI, and LVEDVI in the present study. In addition to the wall extension of the left ventricle, there are many factors, including the age, sex, inflammation, anemia, and renal function, that influence the BNP level^25–28^. In particular, the BNP levels in obese patients with heart failure are lower than those in non-obese patients with heart failure, although obesity is a major risk factor for the development of heart failure^29^. In the patients with heart failure, neuronal hormones, such as catecholamine and NPs, are upregulated and activate lipolysis^30^. In obese cases, even if heart failure is severe, lipolysis is unlikely to occur due to the low level of BNP, and as a result, the production of ketone bodies may be suppressed. Based on these results, the production of ketone bodies is presumed to be more strongly affected by NP levels rather than by the catecholamine levels in patients with heart failure.

Previous studies reported that both plasma BNP levels and blood ketone body levels increase with the aggravation of heart failure^3,4,10,11^. In this study, using a covariance structure analysis, we showed a significant direct correlation between the plasma BNP levels and blood ketone body levels. Although the molecular mechanism by which BNP directly increases the production of ketone bodies is unclear, it is speculated that NPs stimulate lipolysis in adipose tissue and increase free fatty acids (FFAs), which in turn further stimulates the production of ketone bodies in the liver^31,32^.

Our previous studies showed that even very low or physiological levels of BNP exerted various effects on the body^33,34^. In addition, it was shown in the present study that even when BNP levels were normal, the plasma level of BNP contributed to an increase in blood TKB levels. These results proved that the blood TKB levels are not increased by the deterioration of hemodynamics but rather by the physiological effect of BNP, and even a physiological level of BNP might induce the utilization of ketone bodies.

Marcondes-Braga et al. reported that the exhaled breath acetone (EBA) levels differ significantly regarding the function of the severity of heart failure (New York Heart Association [NYHA] classification), and the EBA and BNP levels are also said to be positively correlated^35^. However, they excluded patients who were not using β-blockers and those with diabetes, chronic renal failure, and long-term users of corticosteroids, as these comorbidities and drugs are likely to directly interfere with the ketone body metabolism. In addition, Yokokawa et al. reported that the EBA levels were positively correlated with the NYHA classification and the plasma BNP levels, and EBA significantly correlated with the pulmonary capillary wedge pressure (PCWP)^36^. They also excluded patients with factors that might influence ketone body metabolism, including ischemic heart disease, diabetes, dyslipidemia, chronic hepatitis, end-stage renal failure, and a history of the long-term administration of corticosteroids, tricyclic antidepressants, tetracyclic antidepressants, or selective serotonin reuptake inhibitors. In the present study, we excluded patients who orally took sodium glucose cotransporter (SGLT) 2 inhibitor because it obviously affected the levels of TKB^37–39^. We included other patients with comorbidities and drugs that were likely to influence ketone body metabolism, including—but not limited to—diabetes, dyslipidemia, chronic hepatitis, β-blockers, and corticosteroids. Many patients with heart failure have diabetes and dyslipidemia, and ischemic heart disease is one of the main causes of the onset of heart failure^40,41^. Thus, it cannot be denied that excluding these patients may have resulted in our overlooking essential factors that affect ketone body metabolism. For that reason, we performed covariant structure analyses to clarify the direct contribution of factors, including the BNP, LVEDP, LVESVI, and LVEDVI, to the blood TKB levels and to exclude the effect of confounding factors.

The present study was associated with several limitations. First, this study was conducted at a single university hospital, and the data were collected retrospectively. Second, intrinsically, in order to determine whether NP or catecholamine is involved in lipolysis, the FFA levels—rather than the ketone body levels— should be directly measured; however, in this study, the FFA levels could not be measured due to a lack of reagents. Third, to examine the utilization of ketone bodies in the heart, it is necessary to perform blood sampling at both the aortic root and the coronary sinus simultaneously. In this study, we performed blood sampling from an arterial catheter alone. However, some previous reports have indicated that the uptake of the cardiac ketone bodies increased in proportion to the circulating levels of ketone bodies^6–8^. Fourth, patients with various cardiovascular diseases were included in this study. Not all patients showed apparent left ventricular (LV) systolic dysfunction and/or a substantial increase in plasma BNP levels. Some patients in Stage B according to the Guidelines of Heart Failure^42,43^ were also included. Finally, although this study demonstrated that the blood TKB levels were increased in proportion to the BNP levels, it will be necessary to elucidate through animal experiments the molecular biological mechanism by which the administration of NPs increases blood ketone body levels and to determine whether or not these increased ketone bodies are effectively utilized as energy substrates in the heart.

In conclusion, in patients with cardiovascular disorders, the blood TKB levels were positively increased along with the BNP levels, but not the hemodynamic indices, such as the LVEDP, LVESVI, and LVEDVI. Taken together the results of the present findings suggest that the NP secreted from the failing heart might activate the utilization of ketone bodies as an alternative fuel to fatty acid and glucose in the failing heart. Furthermore, especially in obese patients who show reduced BNP levels, the administration of agents that increase NP levels, such as angiotensin receptor neprilysin inhibitor (ARNI) and carperitide, might exert a cardioprotective effect by improving the energy metabolism via an increase in the blood level of ketone bodies, in addition to the original hemodynamic improvement effect.

## Methods

### Patient population

The study population consisted of 1157 patients who were consecutively admitted to our institution with cardiovascular disorders and who underwent elective cardiac catheterization from October 2016 to April 2020. We excluded patients who underwent dialysis (n = 85). Furthermore, we excluded patients taking an oral SGLT2 inhibitor (n = 42), as this medication obviously increases blood TKB levels^37–39^. Finally, we analyzed 1030 patients in the present study.

The study was approved by the medical ethics committee of Jikei University School of Medicine [24–355(7121)]. The Ethics Committee waived the need for informed written consent, since it was a retrospective study. Instead of obtaining informed consent from each patient, we posted a notice about the study design and contact information at a public location in our institution according to our routine ethical regulations.

### Data collection

The clinical characteristics of patients were retrospectively collected from their medical records. Blood samples were collected from an arterial catheter just before cardiac catheterization with the patient in a fasting condition. Hemodynamic data, such as the LVEDP, were collected during cardiac catheterization. The LVESVI and LVEDVI were measured at the time of left ventriculography. Biochemical analyses of the plasma and serum were performed in our hospital’s central laboratory during the study period. To measure the plasma BNP level, blood samples were collected in tubes containing ethylenediaminetetraacetic acid (EDTA) and then immediately centrifuged at 3000 rpm for 5 min at 14 °C. Thereafter, the plasma BNP levels were immediately measured by a chemiluminescent enzyme immunoassay with AIA-CL2400 (TOSOH Corporation, Tokyo, Japan). The upper normal limit for the plasma BNP level was set at 18.4 pg/mL^42^. The blood TKB level was measured by an enzyme cycling method using TKB-L test kit “Kainos” (KAINOS Laboratories, Inc., Tokyo, Japan).

### Statistical analyses

Data are expressed as the mean ± standard deviation (SD) or as the median (25th, 75th percentile) for significantly skewed variables. Correlations between the clinical parameters and the TKB levels were assessed using Spearman’s rank correlation coefficient. All statistical analyses were performed using the SPSS Statistics software program (version 27.0, SPSS Inc., Chicago, IL, USA). *P* values of < 0.05 were considered to indicate statistical significance.

A path model based on a covariance structure analysis was proposed to investigate the relationships among clinical factors and specifically to identify probable causal effects of the TKB levels. The causality model defined some hierarchical regression models between clinical factors and the blood TKB levels. A path analysis was performed using the IBM SPSS AMOS software program (version 27; Amos Development Corporation, Meadville, PA, USA). The structural equation models that were obtained were tested and confirmed at a significance level of *P* < 0.05.

Furthermore, we applied Bayesian structural equation modeling using a program embedded in IBM SPSS AMOS, version 27 (Amos Development Corporation). The frequency polygon was described with the marginal posterior distributions of the estimands. The selected 2-D contour line was used in this study because it was easily visualized^22,34,44^.

## Supplementary information

Supplementary information.

## References

[1] Kashiwagi Y (2015). Expression of SGLT1 in human hearts and impairment of cardiac glucose uptake by phlorizin during ischemia-reperfusion injury in mice. PLoS ONE.

[2] Nagoshi T, Yoshimura M, Rosano GM, Lopaschuk GD, Mochizuki S (2011). Optimization of cardiac metabolism in heart failure. Curr. Pharm. Des..

[3] Bedi KC (2016). Evidence for intramyocardial disruption of lipid metabolism and increased myocardial ketone utilization in advanced human heart failure. Circulation.

[4] Aubert G (2016). The failing heart relies on ketone bodies as a fuel. Circulation.

[5] Ho KL (2019). Increased ketone body oxidation provides additional energy for the failing heart without improving cardiac efficiency. Cardiovasc. Res..

[6] Fukao T, Lopaschuk GD, Mitchell GA (2004). Pathways and control of ketone body metabolism: on the fringe of lipid biochemistry. Prostaglandins Leukot. Essent. Fatty Acids.

[7] Puchalska P, Crawford PA (2017). Multi-dimensional roles of ketone bodies in fuel metabolism, signaling, and therapeutics. Cell Metab..

[8] Mizuno Y (2017). The diabetic heart utilizes ketone bodies as an energy source. Metab. Clin. Exp..

[9] Lommi J (1996). Blood ketone bodies in congestive heart failure. J. Am. Coll. Cardiol..

[10] Yoshimura M (1993). Different secretion patterns of atrial natriuretic peptide and brain natriuretic peptide in patients with congestive heart failure. Circulation.

[11] Yasue H (1994). Localization and mechanism of secretion of B-type natriuretic peptide in comparison with those of A-type natriuretic peptide in normal subjects and patients with heart failure. Circulation.

[12] Yoshida J (2017). Associations between left ventricular cavity size and cardiac function and overload determined by natriuretic peptide levels and a covariance structure analysis. Sci. Rep..

[13] Kawai M (2013). Determination of the B-type natriuretic peptide level as a criterion for abnormalities in Japanese individuals in routine clinical practice: The J-ABS Multi-Center Study (Japan Abnormal BNP Standard). Int. Med. (Tokyo, Japan).

[14] Yoshimura M, Yasue H, Ogawa H (2001). Pathophysiological significance and clinical application of ANP and BNP in patients with heart failure. Can. J. Physiol. Pharmacol..

[15] Brunner-La Rocca HP, Kaye DM, Woods RL, Hastings J, Esler MD (2001). Effects of intravenous brain natriuretic peptide on regional sympathetic activity in patients with chronic heart failure as compared with healthy control subjects. J. Am. Coll. Cardiol..

[16] Miyashita K (2009). Natriuretic peptides/cGMP/cGMP-dependent protein kinase cascades promote muscle mitochondrial biogenesis and prevent obesity. Diabetes.

[17] Bordicchia M (2012). Cardiac natriuretic peptides act via p38 MAPK to induce the brown fat thermogenic program in mouse and human adipocytes. J. Clin. Investig..

[18] Moro C, Lafontan M (2013). Natriuretic peptides and cGMP signaling control of energy homeostasis. Am. J. Physiol. Heart Circ. Physiol..

[19] Inoue Y (2016). The impact of an inverse correlation between plasma B-type natriuretic peptide levels and insulin resistance on the diabetic condition in patients with heart failure. Metab. Clin. Exp..

[20] Kinoshita K (2016). Potent influence of obesity on suppression of plasma B-type natriuretic peptide levels in patients with acute heart failure: An approach using covariance structure analysis. Int. J. Cardiol..

[21] Kimura H (2017). The thermogenic actions of natriuretic peptide in brown adipocytes: The direct measurement of the intracellular temperature using a fluorescent thermoprobe. Sci. Rep..

[22] Kang R (2020). Possible association between body temperature and B-type natriuretic peptide in patients with cardiovascular diseases. J. Cardiac Fail..

[23] Kashiwagi Y (2020). Therapeutic hypothermia after cardiac arrest increases the plasma level of B-type natriuretic peptide. Sci. Rep..

[24] Abdul Kadir A, Clarke K, Evans RD (2020). Cardiac ketone body metabolism. Biochim. Biophys. Acta Mol. Basis Dis..

[25] Redfield MM (2002). Plasma brain natriuretic peptide concentration: Impact of age and gender. J. Am. Coll. Cardiol..

[26] Harada E (1999). Effect of interleukin-1 beta on cardiac hypertrophy and production of natriuretic peptides in rat cardiocyte culture. J. Mol. Cell. Cardiol..

[27] Tominaga M (2019). Association between plasma B-type natriuretic peptide and anaemia in heart failure with or without ischaemic heart disease: A retrospective study. BMJ Open.

[28] Takami Y (2004). Diagnostic and prognostic value of plasma brain natriuretic peptide in non-dialysis-dependent CRF. Am. J. Kidney Dis..

[29] Mehra MR (2004). Obesity and suppressed B-type natriuretic peptide levels in heart failure. J. Am. Coll. Cardiol..

[30] Lafontan M (2008). Control of lipolysis by natriuretic peptides and cyclic GMP. Trends Endocrinol. Metab. TEM.

[31] McGarry JD, Foster DW (1977). Hormonal control of ketogenesis. Biochemical considerations. Arch. Intern. Med..

[32] Carper D (2020). Atrial natriuretic peptide orchestrates a coordinated physiological response to fuel non-shivering thermogenesis. Cell Rep..

[33] Sugawa S, Masuda I, Kato K, Yoshimura M (2018). Increased levels of cardiac troponin I in subjects with extremely low B-type natriuretic peptide levels. Sci. Rep..

[34] Hasegawa J (2020). Evaluation of enhanced lipid oxidation and compensatory suppression using natriuretic peptide in patients with cardiovascular diseases. Peptides.

[35] Marcondes-Braga FG (2012). Exhaled acetone as a new biomaker of heart failure severity. Chest.

[36] Yokokawa T (2016). Exhaled acetone concentration is related to hemodynamic severity in patients with non-ischemic chronic heart failure. Circ. J..

[37] Taylor SI, Blau JE, Rother KI (2015). SGLT2 inhibitors may predispose to ketoacidosis. J. Clin. Endocrinol. Metab..

[38] Mudaliar S, Alloju S, Henry RR (2016). Can a shift in fuel energetics explain the beneficial cardiorenal outcomes in the EMPA-REG OUTCOME study? A unifying hypothesis. Diabetes Care.

[39] Ferrannini E, Mark M, Mayoux E (2016). CV protection in the EMPA-REG OUTCOME Trial: A "Thrifty Substrate" hypothesis. Diabetes Care.

[40] Ho KK, Pinsky JL, Kannel WB, Levy D (1993). The epidemiology of heart failure: The Framingham Study. J. Am. Coll. Cardiol..

[41] Levy D, Larson MG, Vasan RS, Kannel WB, Ho KK (1996). The progression from hypertension to congestive heart failure. JAMA.

[42] Tsutsui H (2019). JCS 2017/JHFS 2017 guideline on diagnosis and treatment of acute and chronic heart failure-digest version. Circ. J..

[43] Yancy CW (2013). 2013 ACCF/AHA guideline for the management of heart failure: A report of the American College of Cardiology Foundation/American Heart Association Task Force on practice guidelines. Circulation.

[44] Yamada T (2020). Increase in oxidized low-density lipoprotein level according to hyperglycemia in patients with cardiovascular disease: A study by structure equation modeling. Diabetes Res. Clin. Pract..

