# Supplementary material for: Close linkage between blood total ketone body levels and B-type natriuretic peptide levels in patients with cardiovascular disorders
Source: Sci Rep. 2021 Mar 22;11:6498. doi: 10.1038/s41598-021-86126-0 (PMC7985483; doi:10.1038/s41598-021-86126-0)

**Supplementary Information**

**Close linkage between blood total ketone body levels and B-type natriuretic peptide levels in patients with cardiovascular disorders**

Yusuke Kashiwagi* ^1^, Tomohisa Nagoshi^1^, Yasunori Inoue^1^, Yoshiro Tanaka^1^, Hirotake Takahashi^1^, Yuhei Oi^1^, Haruka Kimura^1^, Kousuke Minai^1^, Michihiro Yoshimura^1^

^1^ Division of Cardiology, Department of Internal Medicine, The Jikei University School of Medicine, Tokyo, Japan

**Supplementary Table S1:** The results of the path model (C). (BNP≤18.4 pg/mL) (n=244)

|  |  |  | Estimate | Standard error | Test statistic | P-value | Standardized regression coefficient |
| --- | --- | --- | --- | --- | --- | --- | --- |
|  |  |  |  |  |  |  | Direct effect |
| TKB  (R^2^=0.213) | <--- | Male | 108.598 | 54.042 | 2.010 | 0.044 | 0.185 |
|  | <--- | Age | 1.167 | 2.034 | 0.574 | .0566 | 0.076 |
|  | <--- | BMI | 6.195 | 5.673 | 1.092 | 0.275 | 0.118 |
|  | <--- | Mean blood pressure | -2.580 | 1.651 | -1.563 | 0.118 | -0.214 |
|  | <--- | Heart rate | 6.776 | 4.449 | 1.523 | 0.128 | 0.424 |
|  | <--- | Hb | 7.273 | 14.680 | 0.495 | 0.620 | 0.051 |
|  | <--- | eGFR | -0.559 | 1.023 | -0.546 | 0.585 | -0.047 |
|  | <--- | Total Bilirubin | -4.145 | 83.084 | -0.050 | 0.960 | -0.007 |
|  | <--- | Uric acid | -3.786 | 13.640 | -0.278 | 0.781 | -0.024 |
|  | <--- | CRP | -17.992 | 41.301 | -0.436 | 0.663 | -0.045 |
|  | <--- | Fasting blood sugar | -1.658 | 0.847 | -1.957 | 0.050 | -0.266 |
|  | <--- | HbA1c | 19.329 | 29.447 | 0.656 | 0.512 | 0.081 |
|  | <--- | HDL | 1.025 | 1.073 | 0.955 | 0.339 | 0.084 |
|  | <--- | LDL | 1.036 | 0.650 | 1.595 | 0.111 | 0.151 |
|  | <--- | Triglyceride | -0.815 | 0.234 | -3.486 | ＜0.001 | -0.297 |
|  | <--- | BNP | 5.892 | 2.979 | 1.978 | 0.048 | 0.151 |
|  | <--- | LVEDP | 1.202 | 3.797 | 0.316 | 0.752 | 0.029 |
|  | <--- | LVESVI | -5.148 | 2.883 | -1.785 | 0.074 | -0.241 |
|  | <--- | LVEDVI | 3.870 | 2.585 | 1.497 | 0.134 | 0.309 |

R^2^, squared multiple correlation; TKB, total ketone body; BMI, body mass index; Hb, hemoglobin; eGFR, estimated glomerular filtration rate; CRP, C-reactive protein; HDL, high-density lipoprotein cholesterol; LDL, low-density lipoprotein cholesterol; BNP, B-type natriuretic peptide; LVEDP, left ventricular end-diastolic pressure; LVESVI, left ventricular end-systolic volume index; LVEDVI, left ventricular end-diastolic volume index.

**Supplementary Table S2:** The results of the path model (D). (BNP≤18.4 pg/mL) (n=244)

|  |  |  | Estimate | Standard error | Test statistic | P-value |
| --- | --- | --- | --- | --- | --- | --- |
| TKB  (R^2^=0.122) | <--- | Male | 60.575 | 36.172 | 1.675 | 0.094 |
|  | <--- | Triglyceride | -0.699 | 0.172 | -4.072 | ＜0.001 |
|  | <--- | BNP | 4.941 | 2.453 | 2.014 | 0.044 |
|  | <--- | LVEDP | -1.164 | 2.716 | -0.429 | 0.668 |
|  | <--- | LVESVI | -4.044 | 2.455 | -1.647 | 0.099 |
|  | <--- | LVEDVI | 1.214 | 1.437 | 0.845 | 0.398 |

R^2^, squared multiple correlation; TKB, total ketone body; BNP, B-type natriuretic peptide; LVEDP, left ventricular end-diastolic pressure; LVESVI, left ventricular end-systolic volume index; LVEDVI, left ventricular end-diastolic volume index.

**Supplementary Table S3:** The results of the path model (E). (BNP>18.4 pg/mL) (n=786)

|  |  |  | Estimate | Standard error | Test statistic | P-value | Standardized regression coefficient |
| --- | --- | --- | --- | --- | --- | --- | --- |
|  |  |  |  |  |  |  | Direct effect |
| TKB  (R^2^=0.164) | <--- | Male | -4.251 | 31.482 | -0.135 | 0.893 | -0.005 |
|  | <--- | Age | -0.785 | 1.285 | -0.611 | 0.541 | -0.026 |
|  | <--- | BMI | -4.382 | 3.547 | -1.236 | 0.217 | -0.050 |
|  | <--- | Mean blood pressure | -0.917 | 0.818 | -1.121 | 0.262 | -0.043 |
|  | <--- | Heart rate | 0.198 | 1.276 | 0.155 | 0.877 | 0.009 |
|  | <--- | Hb | -12.122 | 7.826 | -1.549 | 0.121 | -0.072 |
|  | <--- | eGFR | 0.851 | 0.677 | 1.257 | 0.209 | 0.051 |
|  | <--- | Total Bilirubin | 56.801 | 38.536 | 1.474 | 0.140 | 0.066 |
|  | <--- | Uric acid | 11.521 | 7.767 | 1.483 | 0.138 | 0.055 |
|  | <--- | CRP | 56.162 | 9.987 | 5.623 | <0.001 | 0.198 |
|  | <--- | Fasting blood sugar | -0.818 | 0.497 | -1.647 | 0.100 | -0.072 |
|  | <--- | HbA1c | 49.835 | 16.517 | 3.017 | 0.003 | 0.133 |
|  | <--- | HDL | 1.855 | 0.888 | 2.089 | 0.037 | 0.081 |
|  | <--- | LDL | 1.335 | 0.424 | 3.149 | 0.002 | 0.114 |
|  | <--- | Triglyceride | -1.041 | 0.231 | -4.499 | ＜0.001 | -0.172 |
|  | <--- | BNP | 0.197 | 0.065 | 3.021 | 0.003 | 0.172 |
|  | <--- | LVEDP | 0.948 | 2.714 | 0.349 | 0.727 | 0.016 |
|  | <--- | LVESVI | -0.399 | 1.698 | -0.235 | 0.814 | -0.034 |
|  | <--- | LVEDVI | -0.071 | 1.419 | -0.050 | 0.960 | -0.007 |

R^2^, squared multiple correlation; TKB, total ketone body; BMI, body mass index; Hb, hemoglobin; eGFR, estimated glomerular filtration rate; CRP, C-reactive protein; HDL, high-density lipoprotein cholesterol; LDL, low-density lipoprotein cholesterol; BNP, B-type natriuretic peptide; LVEDP, left ventricular end-diastolic pressure; LVESVI, left ventricular end-systolic volume index; LVEDVI, left ventricular end-diastolic volume index.

**Supplementary Table S4:** The results of the path model (F). (BNP>18.4 pg/mL) (n=786)

|  |  |  | Estimate | Standard error | Test statistic | P-value |
| --- | --- | --- | --- | --- | --- | --- |
| TKB  (R^2^=0.147) | <--- | CRP | 59.310 | 9.729 | 6.096 | <0.001 |
|  | <--- | HbA1c | 27.553 | 12.822 | 2.149 | 0.027 |
|  | <--- | HDL | 1.840 | 0.833 | 2.208 | 0.027 |
|  | <--- | LDL | 1.156 | 0.413 | 2.799 | 0.005 |
|  | <--- | Triglyceride | -1.130 | 0.221 | -5.111 | ＜0.001 |
|  | <--- | BNP | 0.135 | 0.065 | 2.073 | 0.038 |
|  | <--- | LVEDP | -0.698 | 2.529 | -0.276 | 0.783 |
|  | <--- | LVESVI | 0.506 | 1.562 | 0.324 | 0.746 |
|  | <--- | LVEDVI | -0.039 | 1.316 | -0.030 | 0.976 |

R^2^, squared multiple correlation; TKB, total ketone body; CRP, C-reactive protein; HDL, high-density lipoprotein cholesterol; LDL, low-density lipoprotein cholesterol; BNP, B-type natriuretic peptide; LVEDP, left ventricular end-diastolic pressure; LVESVI, left ventricular end-systolic volume index; LVEDVI, left ventricular end-diastolic volume index.

**Supplementary Figure Legends**

**Supplementary Figure S1.** Path model (C) (BNP≤18.4 pg/mL) (n=244).

The path model theoretically proposed to clarify the contribution of each of the factors which are show in Table 2 to TKB. Each path has a coefficient representing the standardized coefficient of a regressing independent variable on a dependent variable of the relevant path. These variables represent the standardized regression coefficients (direct effect) (shown in Table S1) and squared multiple correlations (in narrow italics). BMI, body mass index; Mean BP, mean blood pressure; HR, heart rate; Hb, hemoglobin; eGFR, estimated glomerular filtration rate; T-Bil, total-bilirubin; UA, uric acid; CRP, C-reactive protein; FBS, fasting blood sugar; HDL, high-density lipoprotein cholesterol; LDL, low-density lipoprotein cholesterol; TG, triglyceride; BNP, B-type natriuretic peptide; LVEDP, left ventricular end-diastolic pressure; LVESVI, left ventricular end-systolic volume index; LVEDVI, left ventricular end-diastolic volume index.

**Supplementary Figure S2.** Path model (D) (BNP≤18.4 pg/mL) (n=244).

The path model theoretically proposed to clarify the contribution of each factor (Male, TG, BNP, LVEDP, LVESVI, and LVEDVI) to TKB. Each path has a coefficient representing the standardized coefficient of a regressing independent variable on a dependent variable of the relevant path. These variables represent the standardized regression coefficient (direct effect) (underlined portions indicate remarkable values) and squared multiple correlation (in narrow italics). TG, triglyceride; BNP, B-type natriuretic peptide; LVEDP, left ventricular end-diastolic pressure; LVESVI, left ventricular end-systolic volume index; LVEDVI, left ventricular end-diastolic volume index.

**Supplementary Figure S3.** Bayesian structure equation modeling (BNP≤18.4 pg/mL) (n=244). Frequency polygons were described by the marginal posterior distributions of the estimates. The two-dimensional plot of the bivariate posterior density shows the relationship between the bivariate marginal posterior plots. From light to dark, the 3 shades of gray represent 50%, 90%, and 95% reliable regions, respectively. BNP, B-type natriuretic peptide; LVEDP, left ventricular end-diastolic pressure; LVESVI, left ventricular end-systolic volume index; LVEDVI, left ventricular end-diastolic volume index.

**Supplementary Figure S4.** Path model (E) (BNP>18.4 pg/mL) (n=786).

The path model theoretically proposed to clarify the contribution of each of the factors which are show in Table 2 to TKB. Each path has a coefficient representing the standardized coefficient of a regressing independent variable on a dependent variable of the relevant path. These variables represent the standardized regression coefficients (direct effect) (shown in Table S3) and squared multiple correlations (in narrow italics). BMI, body mass index; Mean BP, mean blood pressure; HR, heart rate; Hb, hemoglobin; eGFR, estimated glomerular filtration rate; T-Bil, total-bilirubin; UA, uric acid; CRP, C-reactive protein; FBS, fasting blood sugar; HDL, high-density lipoprotein cholesterol; LDL, low-density lipoprotein cholesterol; TG, triglyceride; BNP, B-type natriuretic peptide; LVEDP, left ventricular end-diastolic pressure; LVESVI, left ventricular end-systolic volume index; LVEDVI, left ventricular end-diastolic volume index.

**Supplementary Figure S5.** Path model (F) (BNP>18.4 pg/mL) (n=786).

The path model theoretically proposed to clarify the contribution of each factor (CRP, HbA1c, HDL, LDL, TG, BNP, LVEDP, LVESVI and LVEDVI) to TKB. Each path has a coefficient representing the standardized coefficient of a regressing independent variable on a dependent variable of the relevant path. These variables represent the standardized regression coefficient (direct effect) (underlined portions indicate remarkable values) and squared multiple correlation (in narrow italics). CRP, C-reactive protein; HDL, high-density lipoprotein cholesterol; LDL, low-density lipoprotein cholesterol; TG, triglyceride; BNP, B-type natriuretic peptide; LVEDP, left ventricular end-diastolic pressure; LVESVI, left ventricular end-systolic volume index; LVEDVI, left ventricular end-diastolic volume index.

**Supplementary Figure S6.** Bayesian structure equation modeling (BNP>18.4 pg/mL) (n=786). Frequency polygons were described by the marginal posterior distributions of the estimates. The two-dimensional plot of the bivariate posterior density shows the relationship between the bivariate marginal posterior plots. From light to dark, the 3 shades of gray represent 50%, 90%, and 95% reliable regions, respectively. BNP, B-type natriuretic peptide; LVEDP, left ventricular end-diastolic pressure; LVESVI, left ventricular end-systolic volume index; LVEDVI, left ventricular end-diastolic volume index.

**Supplementary Figure S1.** Path model (C) (BNP≤18.4 pg/mL) (n=244).


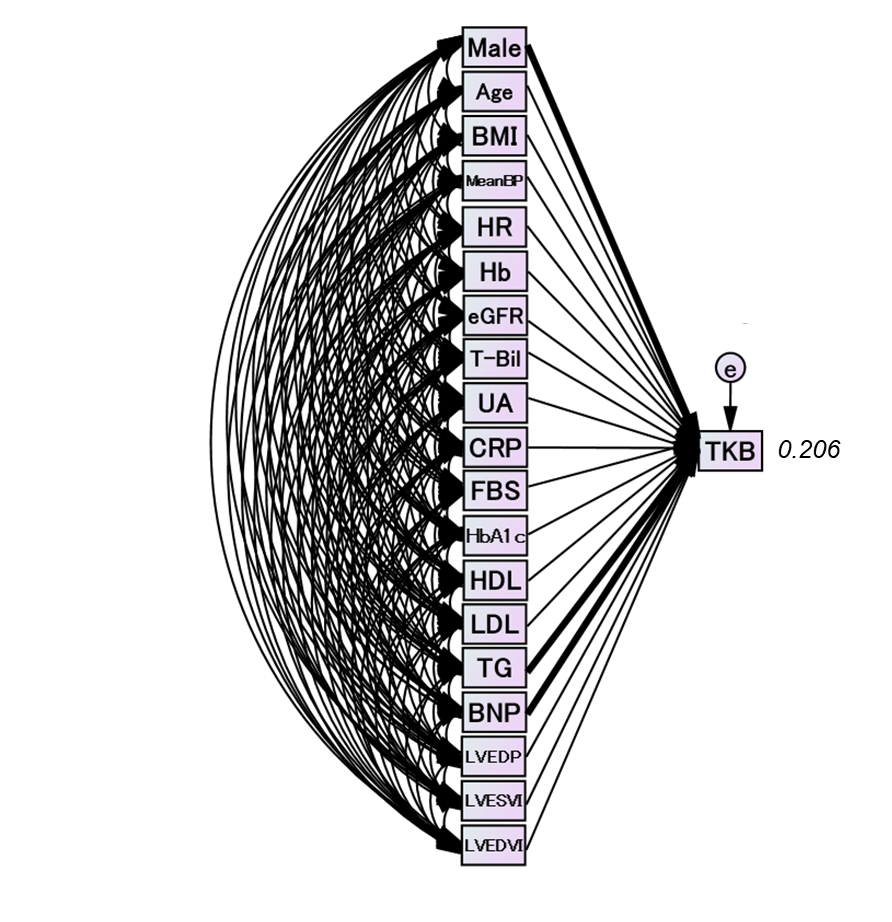


**Supplementary Figure S2.** Path model (D) (BNP≤18.4 pg/mL) (n=244).


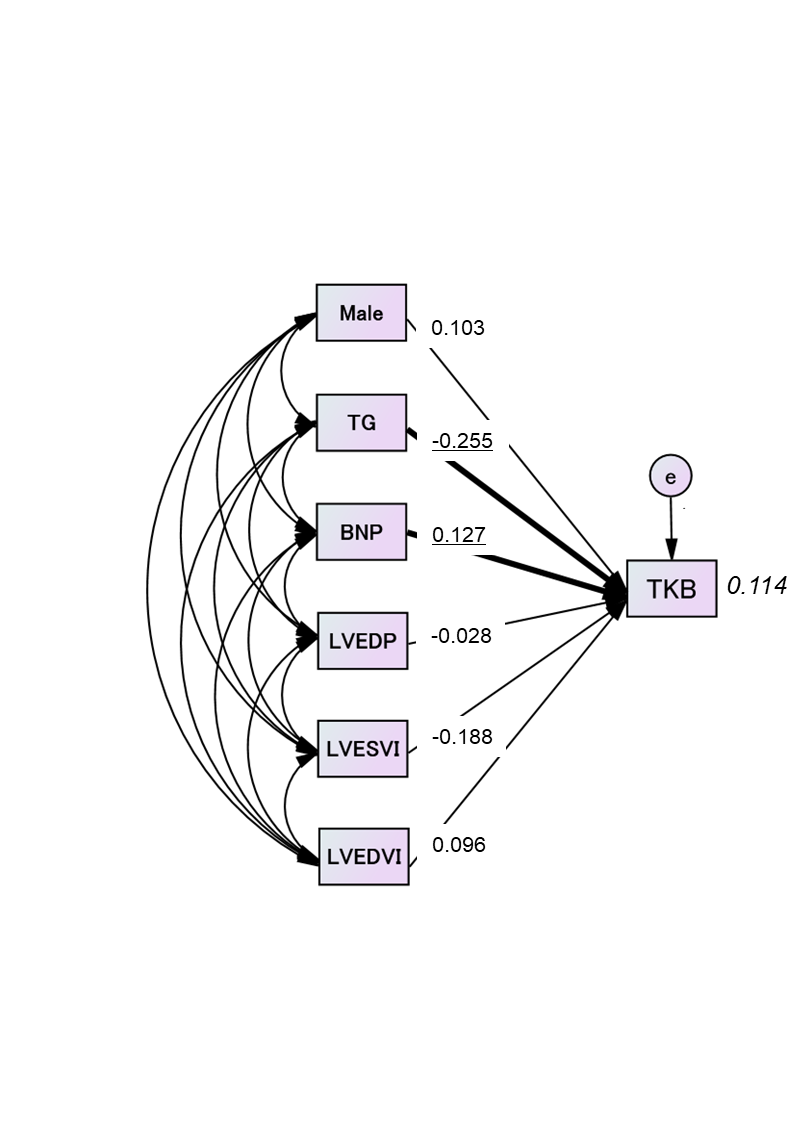


**Supplementary Figure S3.** Bayesian structure equation modeling (BNP≤18.4 pg/mL) (n=244).


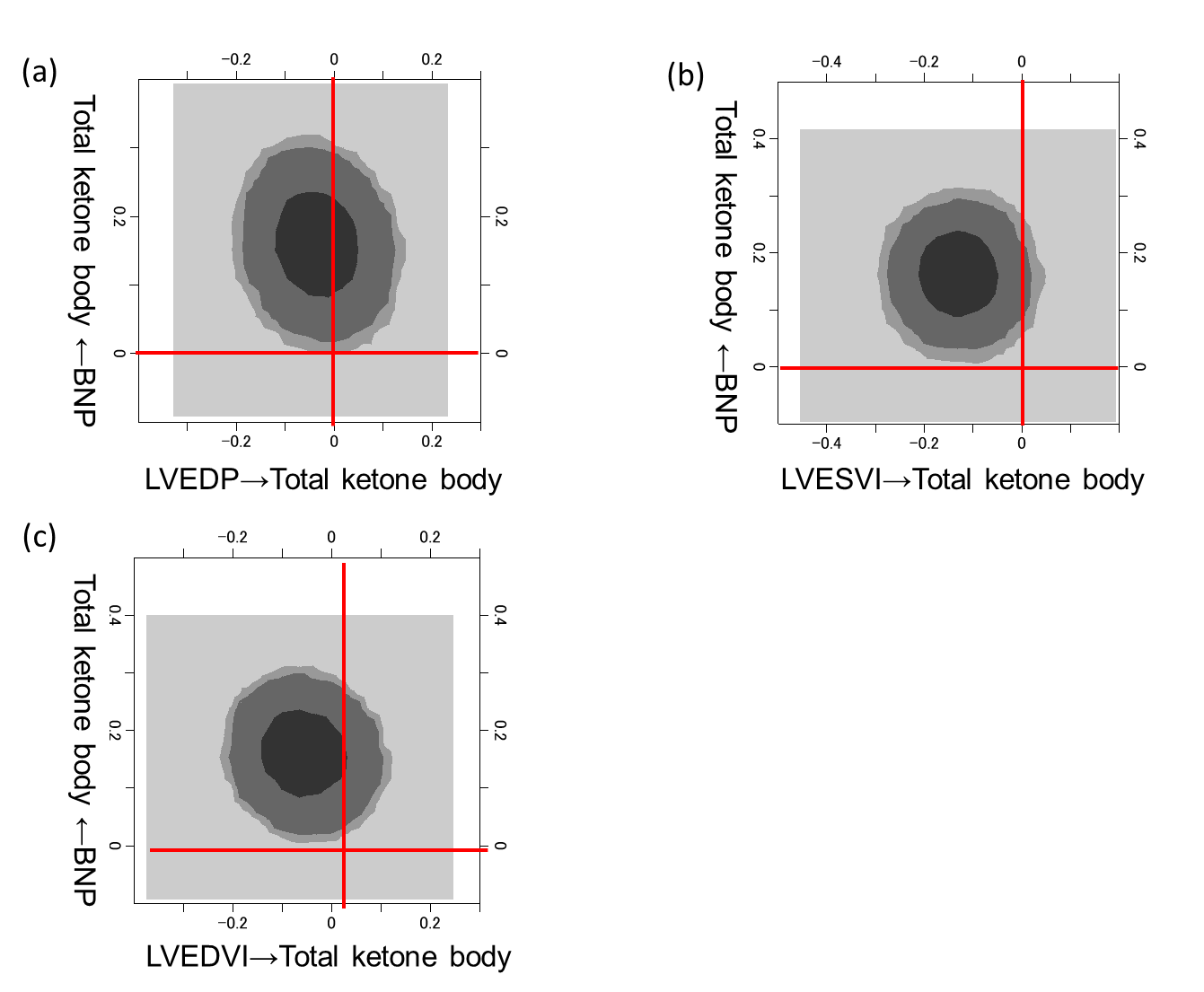


**Supplementary Figure S4.** Path model (E) (BNP >18.4 pg/mL) (n=786).
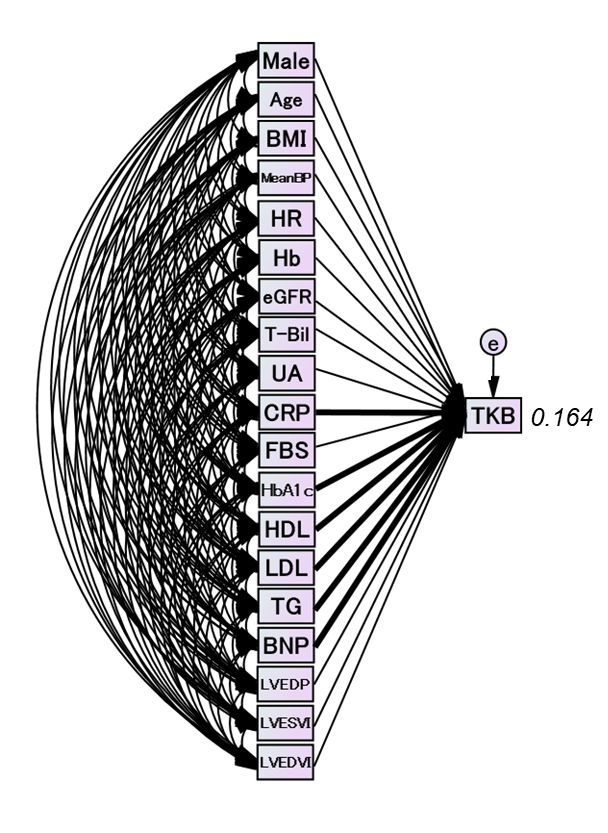


**Supplementary Figure S5.** Path model (F) (BNP >18.4 pg/mL) (n=786).


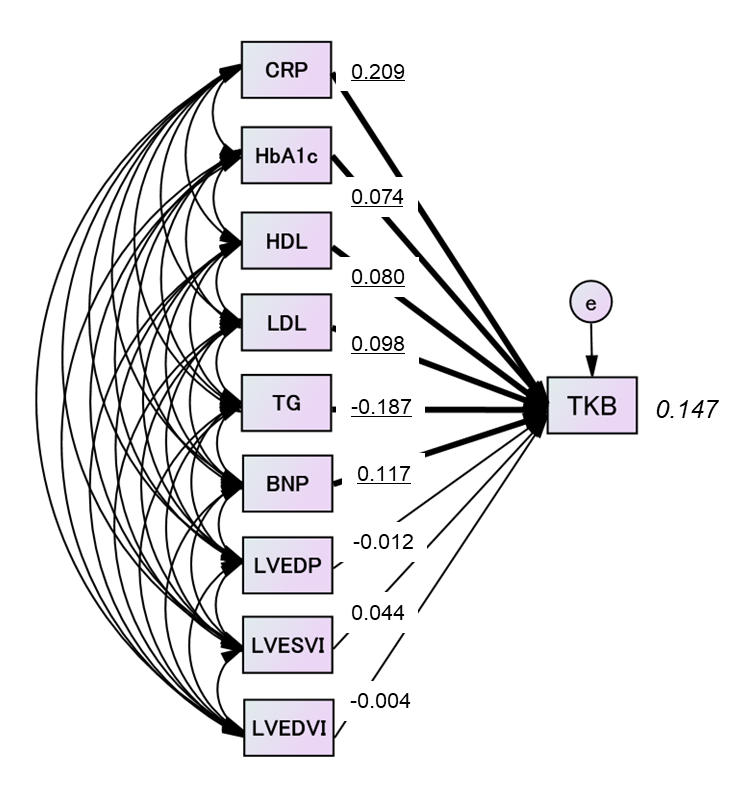


**Supplementary Figure S6.** Bayesian structure equation modeling (BNP >18.4 pg/mL) (n=786).


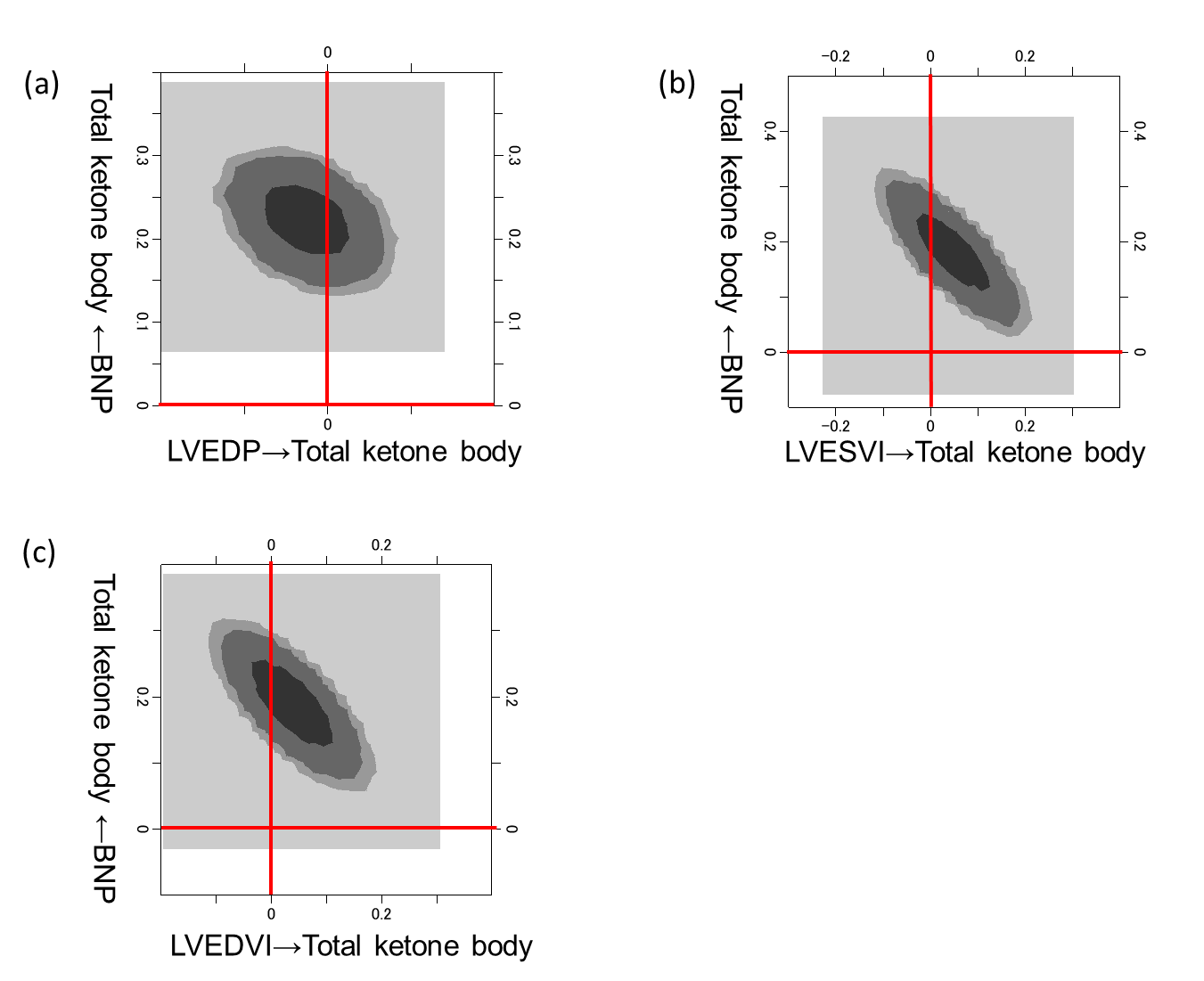

Supplement: Supplementary file 1 — Supplementary information. [file 41598_2021_86126_MOESM1_ESM.docx]
